# Comparing Short-Term Outcomes of Ventral Hernia Repair Using Heavyweight Non-Woven Polypropylene Mesh With Heavyweight Knitted Polypropylene Mesh

**DOI:** 10.3389/jaws.2025.14316

**Published:** 2025-04-04

**Authors:** Aldo Fafaj, Lucas R. A. Beffa, Clayton C. Petro, Ajita S. Prabhu, Benjamin T. Miller, Li-Ching Huang, Ryan C. Ellis, Sara M. Maskal, Nir Messer, Sergio Mazzola Poli de Figueiredo, Michael J. Rosen

**Affiliations:** ^1^ Center for Abdominal Core Health, Digestive Disease and Surgery Institute, Cleveland Clinic Foundation, Cleveland, OH, United States; ^2^ Department of Biostatistics, Vanderbilt University Medical Center, Nashville, TN, United States

**Keywords:** hernia, mesh, mesh complications, abdominal wall reconstruction, hernia repair

## Abstract

**Introduction:**

The mesh choice for the majority of our retromuscular repairs is heavyweight knitted polypropylene (KP) mesh. However, supply chain issues necessitated a change to a newer non-woven polypropylene mesh (NWP). We aimed to evaluate our initial experience with using NWP mesh in retromuscular abdominal wall reconstruction.

**Methods:**

We performed a retrospective review of all patients at our institution who underwent elective, open incisional hernia repair with NWP or KP mesh from January 2014 until December 2023. The analyzed variables included patient demographics, comorbidities, operative techniques, mesh type, position, and postoperative outcomes. A propensity score model and matching algorithms were implemented to address potential treatment-choice bias. Patients receiving NWP mesh were matched with patients receiving KP mesh in a 1:2 ratio.

**Results:**

A total of 771 patients were included in the study, 63 (8.2%) patients had their hernia repaired with NWP and 708 (91.2%) patients with KP mesh. After propensity score matching, 63 patients in the NWP group and 126 in the KP were analyzed. At 30-day follow-up, there were significantly more deep SSIs in the NWP group, however, there were no differences in readmission, reoperation, hernia recurrence, and overall SSI, SSO, and SSOPI.

**Conclusion:**

Retromuscular hernia repaired with non-woven polypropylene mesh showed no difference in readmission, reoperation, hernia recurrence, and overall SSI, SSO, and SSOPI when compared with knitted polypropylene. There were significantly more deep SSIs in the NWP group; however, in all cases, the mesh was salvaged with local wound care, and all patients made a complete recovery. In the short term, the use of NWP mesh appears to be safe, with outcomes comparable to KP mesh.

## Introduction

Mesh is widely accepted as the best approach to hernia repair since it was shown to reduce recurrence rates [1]. Our group recently published a large, multicenter randomized controlled trial which showed that heavyweight KP mesh had similar outcomes compared to mediumweight KP mesh in terms of wound morbidity, patient-reported quality of life, and patient perception of the prosthetic [2]. Given these results, coupled with the evidence of central mesh failure, our group started to routinely utilize heavyweight KP for most of our retromuscular hernia repairs.

In 2022, due to supply chain issues, our heavyweight KP mesh vendor was unable to provide this mesh, so this necessitated a change to a newer non-woven heavyweight polypropylene mesh (NWP), brand name SURGIMESH^®^. When compared with KP, which is typically a monofilament knitted scaffold, NWP mesh has some unique properties utilizing very small polypropylene fibers laid in place in random patterns [3]. Many commercially available knitted meshes are manufactured through a warp-knitting process by feeding multiple individual yarns from warp beams onto specialized knitting needles on a warp-knitting machine, where the yarns are interlocked in a specific pattern to create a mesh, using a medical-grade polymer like monofilament polypropylene. In contrast, non-woven meshes are manufactured through electrospinning, a spinning technique that uses electrostatic forces to produce fibrous scaffolds from biocompatible polymers, such as polypropylene. Electrospun non-wovens exhibit a high surface-to-volume ratio, porosity, pore interconnectivity, and other easy-tailorable properties. The ECM-like, three-dimensional architecture is thought to support cellular adhesion, spreading, and functions, while the intrinsic porosity and pore interconnectivity facilitate angiogenesis, ultimately promoting tissue homeostasis and repair [3]. Herein, we compare our initial experience using NWP heavy-weight mesh in retromuscular abdominal wall reconstruction compared to heavyweight KP.

## Methods

After approval from the Institutional Review Board, the patients were identified using the Abdominal Core Health Quality Collaborative (ACHQC). This prospective, surgeon-entered quality improvement effort aims to improve outcomes through sharing transparent data and collaborative learning. The information is prospectively collected using standardized definitions for preoperative, operative, and post-operative phases of care. Details regarding the registry’s design, implementation, and data quality assurance have been previously published [4].

The study population included all patients at our institution who underwent open ventral hernia repair with NWP or KP mesh from January 2014 until December 2023 and who had 30-day follow-up available at the outpatient clinic. We elected to study only open cases to minimize confounding factors. Similarly, the mesh size for inclusion in this comparison was limited to up to 30 cm by 30 cm for both arms because hernias repaired with larger-sized mesh are, by definition, more complex and could confound the results. A retrospective review of the prospectively collected data was then performed. The variables analyzed included patient demographics, comorbidities, and the operative technique, including mesh type, position, and postoperative outcomes. Our outcomes of interest were 30-day wound morbidities and post-operative wound events, including surgical site infection (SSI), surgical site occurrence (SSO), and SSO requiring procedural intervention (SSOPI) [5, 6]. SSI was classified as superficial, deep, or organ space according to the Centers for Disease Control and Prevention (CDC) standards. SSO included all SSI, in addition to wound cellulitis, non-healing incisional wound, fascial disruption, skin or soft tissue ischemia, skin or soft tissue necrosis, serous or purulent wound drainage, stitch abscess, seroma, hematoma, infected or exposed mesh, or development of an enterocutaneous fistula. Procedural interventions to be considered SSOPI included wound opening, wound debridement, suture excision, percutaneous drainage, partial mesh removal, and complete mesh removal. A propensity score model and matching algorithms were then implemented to address potential treatment-choice bias. Propensity score matches (PSM) were generated by matching patients receiving NWP mesh with patients receiving KP mesh. Two matched controls were selected for each case. A logistic regression model was used to estimate the propensity scores. The model included gender, BMI, COPD, smoking, immunosuppression, history of abdominal wall SSI, prior prosthetic mesh infection, hernia width, hernia length, wound status, hernia recurrent, and age. These variables were selected based on clinical considerations. Due to the low missing rate (<1%), only complete cases were included in the PSM analysis. Nearest neighbor matching without replacement was used to match the patients. No caliper was used to keep all of the NWP patients in the analysis. The standardized mean differences (SMD) were used to evaluate the balance between two mesh types pre- and post-matching [7]. The SMD less than 0.2 was considered acceptable however, values less than or around 0.1 indicate good balance. Two-sided p-values less than or equal to 0.05 were considered statistically significant. All analyses were performed using R 4.2 in addition to R packages: Hmisc, rms, MatchIt, tableone, and survey.

## Results

**FIGURE 1:**
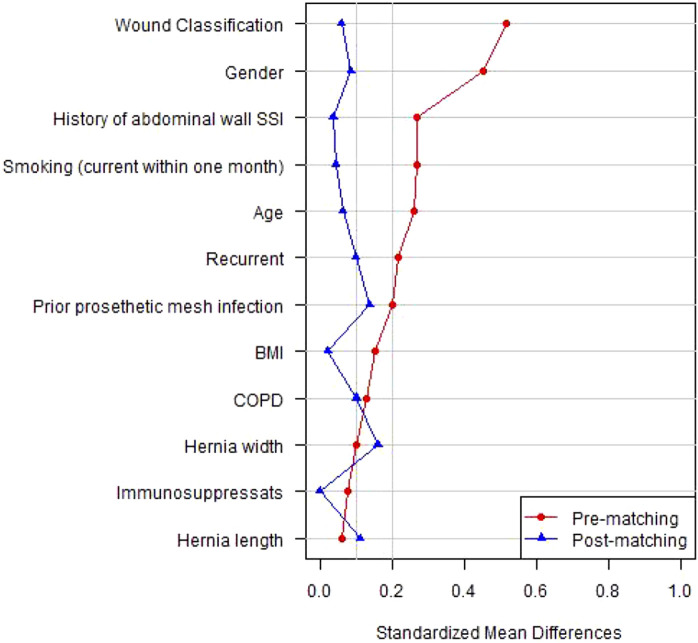
Standardized mean difference (SMD) of several baseline covariates deemed to be important predictors of wound complications.

A total of 771 patients were included in the study; 63 (8.2%) patients had their hernia repaired with NWP and 708 (91.2%) patients with KP mesh. Unmatched patient demographics for each group are presented in Table 1. The two groups had notable differences in the patient demographics and hernia characteristics. The NWP mesh group had more patients that were females, smokers, ASA class 4, and prior abdominal wall SSI. The NWP group also had more concomitant procedures and clean-contaminated cases. Figure 1 shows the standardized mean difference (SMD) of several baseline covariates deemed to be important predictors of wound complications. The red line indicates the SMD of the cohort without adjustment, and the blue line indicates the SMD after adjustment. The SMD less than 0.2 was considered acceptable however, values less than or around 0.1 indicate good balance. Patient demographics, hernia characteristics and intraoperative details, post-match, revealed two well matched groups, 63 patients in the NWP group and 126 in the KP group which are shown in Table 2. After the match, the KP group had a lower rate of transfascial mesh fixation when compared to the KP group (4.8% vs. 44.4%, p < 0.001), representing a change in our practice based on a recently published randomized controlled trial [8]. All patients had a 30 cm long by 30 cm wide mesh placed, except one patient in the NWP group who had placement of a 15 cm long by 15 cm wide mesh.

**TABLE 1:** Patient and hernia characteristics for the unmatched cohort.

	NWP	KP	P-value
N	63	708	
Age (IQR)	61 (54–68)	59 (50–67)	0.12
Gender, N (%)			
Female	45 (71)	352 (50)	<0.001
BMI, kg/m^2^, (IQR)	35 (29–36)	32 (29–36)	0.37
ASA, N (%)			<0.001
2	2 (3)	92 (13)	
3	55 (87)	604 (85)	
4	6 (10)	12 (2)	
Hypertension, N (%)	50 (79)	441 (62)	0.007
Diabetes Mellitus, N (%)	21 (33)	177 (25)	0.15
COPD, N (%)	8 (13)	61 (9)	0.28
Anti-coagulation medications, N (%)	7 (11)	50 (7)	0.24
Immunosuppressants, N (%)	6 (10)	53 (7)	0.56
Current smoking, N (%)	10 (16)	52 (7%)	0.017
History of open abdomen, N (%)	5 (8)	15 (2)	0.46
History of abdominal wall SSI, N (%)	16 (25)	105 (15)	0.027
Prior prosthetic mesh infection, N (%)	7 (11)	40 (6)	0.083
Wound classification, N (%)			<0.001
Clean	50 (79)	666 (94)	
Clean-contaminated	13 (21)	33 (5)	
Contaminated	0 (0)	8 (1)	
Dirty/Infected	0 (0)	1 (0)	
Hernia width, cm, (IQR)	14 (11–16)	14 (12–16)	0.51
Hernia length, cm, (IQR)	22 (18–25)	22 (18–25)	0.88
Recurrent, N (%)	42 (67)	397 (56)	0.1
Concomitant procedure performed, N (%)	13 (21)	58 (8)	0.001
Myofascial Release, N (%)	63 (100)	703 (99)	0.5

BMI, Body Mass Index; ASA, The American Society of Anesthesiologists Physical Status Classification System; COPD, Chronic Obstructive Pulmonary Disease; IQR, interquartile range.

**TABLE 2:** Patient demographics and hernia characteristics after propensity score matching.

	NWP	KP	P-value
N	63	126	
Age, (IQR)	61 (54–68)	63 (53–70)	0.74
Gender, N (%)			1
Female	45 (71)	90 (71)	
BMI, kg/m^2^, (IQR)	35 (29–36)	33 (29–37)	0.71
COPD, N (%)	8 (13)	17 (13)	0.88
Current smoking, N (%)	10 (16)	20 (16)	1
Immunosuppressants, N (%)	6 (10)	9 (7)	0.57
History of abdominal wall SSI, N (%)	16 (25)	31 (25)	0.90
Prior prosthetic mesh infection, N (%)	7 (11)	11 (9)	0.6
Recurrent, N (%)	42 (67)	81 (64)	0.75
Wound classification, N (%)			0.5
Clean	50 (79)	105 (83)	
Clean-contaminated	13 (21)	21 (17)	
Contaminated	0 (0)	0 (0)	
Dirty/Infected	0 (0)	0 (0)	
Hernia width, cm, (IQR)	14 (11–15.5)	14 (12–15)	0.94
Hernia length, cm, (IQR)	22 (17.5–25)	23 (22–25)	0.3
Mesh location, N (%)			0.16
Onlay	0	0	
Inlay	0	0	
Sublay	63	126	
Myofascial Release, N (%)			0.48
Yes	63 (100)	125 (99.2)	
No	0	1 (0.8)	
Transversus abdominis release, N (%)			0.48
Yes	63 (100)	124 (99.2)	
No	0	1 (0.8)	
Fixation used, N (%)			<0.001
Yes	3 (4.8)	56 (44.4)	
No	60 (95.2)	70 (55.6)	
Anterior fascial closure	63 (100)	123 (98%)	0.2

BMI, Body Mass Index; COPD, Chronic Obstructive Pulmonary Disease; IQR, interquartile range.

Post-operative outcomes are shown in Table 3. At 30 days follow-up, there were no differences in readmission, reoperation, hernia recurrence, and overall SSI, SSO, and SSOPI. Notably, there were 16 SSI events during the study period (4 in the NWP group vs. 12 in the KP group, p = 0.46). The majority of SSI in the KP group were superficial (11/12 in the KP group vs. 1/4 in the NWP group, p = 0.008), whereas deep SSI comprised all the SSI in the NWP group and only 1/12 in the KP group (p < 0.001). With regards to the deep SSIs, three out of the four deep SSIs in the NWP group occurred in clean-contaminated cases. Three patients were managed with wet-to-dry packing, with the infection tracking to the anterior fascia but not involving the retromuscular prosthetic, and one patient required an image-guided drain placement for an infected hematoma in the retromuscular space around the prosthetic. All patients received oral antibiotics and the patient requiring drain placement also had IV antibiotics and antibiotic flushes through the drain. There were no mesh excisions, and all four patients completely resolved their infections without further interventions. The patient treated with percutaneous drainage had no signs of infection at 8 months follow up and has retained the mesh. In the KP group, there was one deep SSI. This patient required several takebacks to the operating room for washout, multiple mesh debridements in the office that led to a hernia recurrence, and ultimately underwent a redo abdominal wall reconstruction.

**TABLE 3:** 30-day outcomes.

	NWP	KP	P-value
N	63	126	
Readmission, N (%)	4 (6.3)	10 (7.9)	0.69
Readmission reason			
Wound complication	2 (3.1)	3 (2.4)	
Gastrointestinal complication	2 (3.1)	5 (3.9)	
Bleeding complication	0 (0)	1 (0.8)	
Reoperation, N (%)	0 (0)	2 (1.6)	0.32
Reoperation reason, N (%)			
Major wound complication	0 (0)	1 (0.8)	
Unrelated intra-abdominal pathology	0 (0)	1 (0.8)	
Surgical Site Infection, N (%)	4 (6.3)	12 (9.5)	0.46
Superficial	1 (1.6)	11 (8.7)	0.008
Deep	4 (6.3)	1 (0.8)	<0.001
Organ space	0 (0)	0 (0)	
SSO requiring procedural intervention, N (%)	6 (9.5)	8 (6.3)	0.43
Pulmonary Embolism, N (%)	0 (0)	2 (5.6)	0.2
Urinary tract infection, N (%)	1 (3.4)	2 (5.6)	0.69
Acute renal failure, N (%)	0 (0)	2 (5.6)	0.2
Pneumonia, N (%)	4 (14)	4 (11)	0.74
Respiratory failure requiring intubation, N (%)	1 (3.4)	0 (0)	0.26
Post op bleeding requiring transfusion, N (%)	7 (24)	6 (17)	0.45

SSO, Surgical site occurrence.

## Discussion

This study compared heavyweight non-woven polypropylene (NWP) mesh with heavyweight knitted polypropylene (KP) mesh in abdominal wall reconstruction. After propensity score matching, we found that at 30 days follow-up, when compared to KP mesh, NWP mesh had similar readmission, reoperation, hernia recurrence, and overall SSI and SSOPI rate. Of note, NWP had a higher rate of deep SSI, but all cases resolved with local wound care, and none required any mesh excision.

Our approach to hernia repair, particularly when it comes to mesh choice, has recently changed based on a multicenter randomized controlled trial published in 2021. When looking at the effect of mesh weight on postoperative outcomes in 350 patients, Krpata et al. showed that mediumweight KP mesh did not have any clinical benefits over heavyweight KP mesh [2]. Given evidence of medium-weight mesh fractures, which can be as high as 4.2%, our group switched to using primarily heavyweight polypropylene mesh for clean cases [9]. It must be highlighted that in the study by Krpata et al, all cases were clean, and the rate of SSI were 4.8% in the heavyweight KP mesh group and 5.5% in the mediumweight KP mesh group. In our series, 20% of the cases were clean contaminated, which certainly confounds the results, as indicated by the higher rate of SSI in the current study. We must highlight that this represents a change in our practice overtime as we gained more experience with using heavyweight mesh in clean contaminated cases. The higher rate of SSI in our series remained present when comparing superficial and deep SSIs. There were no organ space infections in either study. Notably, using heavyweight mesh in clean contaminated cases remains controversial and should be further studied with a randomized controlled trial.

There are several unique features of NWP mesh that, at least theoretically, might provide some advantage over KP mesh. To form the NWP mesh, very small (0.02 mm in diameter) polypropylene fibers are randomly oriented and laid in place. Histological evaluations of NWP mesh implanted in animal models have shown planar deposition of connective tissue leading to the formation of collagen, which is primarily oriented in the plane of the surgical mesh and with minimal disruptions in the connective tissue and collagen. In contrast, KP meshes are formed with larger fibers (0.1–0.34 mm in diameter) and have a greater distance between each fiber which leads to connective tissue disruptions. When compared to non-barrier KP in histopathology birefringence analysis, non-barrier NWP had significantly less connective tissue disruptions (0.5% vs. 12.7%, p < 0.0001) [3]. The lower percentage of connective tissue disruptions, coupled with a planar connective tissue orientation, have been theorized as a better approach in mesh design as it may prevent mechanical mesh failures. While we did not notice any significant differences in early performance between NWP and KP, these patients undergo continued surveillance, and eventually, we will evaluate the long-term outcomes of hernia recurrence and patient-reported quality of life.

The difference in deep SSI rates deserves further consideration, particularly regarding whether it was a result of the complexity of the cases or the prosthetic itself. Given the retrospective nature of this study, it is impossible to establish causation. First, 3/4 of cases in the NWP group that had deep SSI were in clean-contaminated cases. Second, according to the CDC guidelines, our definition of deep SSI includes an infection involving the anterior fascia. In 3/4 of the deep SSI, the anterior fascia was exposed but the prosthetic in the retromuscular space was not involved. In the one case involving the prosthetic, a retromuscular hematoma became infected in a patient with a prior MRSA mesh infection who was actively smoking. After percutaneous drainage and antibiotic irrigation as described by Trunzo et al. [10], we were able to salvage the mesh, and the patient remains hernia-free with no signs of mesh infection at 8 months follow-up. In comparison, the only deep SSI in the KP group required several takebacks to the operating room for washout, multiple mesh debridements in the office that led to a hernia recurrence, and ultimately underwent a redo abdominal wall reconstruction. These are a small number of events in relatively small groups, so it is difficult to draw any conclusions. However, the safety and efficacy of heavyweight polypropylene mesh in non-clean cases should be evaluated in a prospective trial.

Although outside the scope of this study, there are other potential advantages to using NWP mesh as it is the only commercially available heavyweight polypropylene mesh that has large mesh sizes, including up to 50 cm × 50 cm. At our institution, the biggest available size of heavyweight KP meshes are 30 cm long by 30 cm wide. These meshes do not provide adequate overlap when dealing with large incisional defects, so several pieces must be sewn together. The concern with this technique was the multitude of permanent sutures needed since these types of sutures have been linked to suture sinus formation [11]. The risk for mesh infection with sewn-together heavyweight KP was often balanced with the risk of mesh fracture if the mediumweight mesh was used, which comes in sizes up to 50 cm long by 50 cm wide. The mesh choice was even more difficult in clinical scenarios when the anterior fascia could not be closed completely, leading to a bridged repair. We know that this challenging cohort has a higher risk for wound morbidity [12], which makes paneled mesh less ideal. Additionally, this group also has a much higher risk for mesh fracture than those who are able to undergo reapproximation of the fascia, up to 30%, when medium-weight KP is used [9]. The long-term outcomes of these newer NWP mesh will need to be evaluated to determine the risk of mesh fracture in these challenging bridging situations.

This study is not without limitations. First, this is a retrospective review of prospectively collected data, so there may be biases associated with this type of study. Second, we limited the mesh sizes to 30 cm wide by 30 cm long in both groups. The reason for this is that the complexity of hernias increases when bigger pieces of mesh are used and that there are no KP meshes bigger than 30 cm wide by 30 cm long available at our institution. As such, the results are not generalizable to bigger mesh sizes. Third, this study looked at short-term outcomes, so we do not know how the NWP mesh compares to KP mesh long-term, especially with regards to hernia recurrence and patient-reported outcomes, so longer follow-up is needed. Finally, there was a statistically significant difference in mesh fixation rates between the two groups, higher rate for the KP group, which could have affected the results. However, this difference is due to our practice change after our randomized controlled trial looking at transfascial mesh fixation for open abdominal wall reconstruction which found that no transfascial fixation was non inferior to transfascial fixation [8]. Importantly, there were no differences in the rates of overall SSI between the two groups or mesh infections requiring mesh removal. As such, we do not believe mesh fixation has any effect on wound infection.

## Conclusion

When compared to heavyweight KP mesh, heavyweight NWP mesh group had no differences in readmission, reoperation, hernia recurrence, and overall SSI, SSO and SSOPI. There were significantly more deep SSIs in the NWP group; however, in all cases, the mesh was salvaged with local wound care, and all patients made a complete recovery. In the short-term, the use of NWP mesh appears to be safe with outcomes comparable to KP mesh for meshes up to 30 by 30 cm. Long-term data are needed to evaluate the use of NWP mesh further. Finally, the use of either mesh in non-clean cases remains experimental, requiring carefully selected patients, and our experience is not generalizable.

## Data Availability

The data analyzed in this study is subject to the following licenses/restrictions: The ACHQC data is available to the ACHQC statisticians who provided the analysis. Requests to access these datasets should be directed to http://.achqc.org.
